# The Effect of Caffeine on Heart Rate Variability in Newborns: A Pilot Study

**DOI:** 10.3390/life13071459

**Published:** 2023-06-27

**Authors:** Helena Lenasi, Eva Rihar, Jerneja Filipič, Matjaž Klemenc, Petja Fister

**Affiliations:** 1 Institute of Physiology, Medical Faculty, University of Ljubljana, Zaloška cesta 4, 1000 Ljubljana, Slovenia; helena.lenasi.ml@mf.uni-lj.si; 2 Children’s Hospital, University Medical Centre Ljubljana, Zaloška cesta 2, 1000 Ljubljana, Slovenia; eva.rihar@kclj.si (E.R.); jerneja.filipic@kclj.si (J.F.); 3 Department of Cardiology, General Hospital Dr. Franc Derganc, Ulica Padlih Borcev 13A, 5290 Šempeter pri Gorici, Slovenia; matjaz.klemenc@bolnisnica-go.si; 4 Children’s Hospital, Pediatric Intensive Care Unit, University Medical Centre Ljubljana, Bohoričeva ulica 20, 1000 Ljubljana, Slovenia

**Keywords:** newborn, heart rate variability, caffeine, apnoea, apnea, autonomic nervous system

## Abstract

Neonatal apnoea can be treated with caffeine, which affects the central nervous and cardiovascular systems. Heart rate variability (HRV) reflects the activity of the autonomic nervous system (ANS) and might be used as a measure of ANS maturation in newborns. We aimed to establish the effect of caffeine on HRV in newborns and investigated the potential correlation between HRV and postmenstrual age (PMA). In 25 haemodynamically stable newborns hospitalized due to apnoea and treated with caffeine (2.5 mg/kg), we assessed breathing frequency, arterial oxygen saturation, body temperature, and the heart rate while they were sleeping. We assessed HRV by spectral analysis using fast Fourier transformation. The same protocol was reapplied 100 h after caffeine withdrawal to assess the control parameters. Caffeine increased breathing frequency (*p* = 0.023) but did not affect any other parameter assessed including HRV. We established a positive correlation between postmenstrual age and HRV during treatment with caffeine as well as after caffeine had been withdrawn (total power: *p* = 0.044; low-frequency band: *p* = 0.039). Apparently, the maintenance dose of caffeine is too low to affect the heart rate and HRV. A positive correlation between PMA and HRV might reflect maturation of the ANS, irrespective of caffeine treatment.

## 1. Introduction

Neonatal apnoea is a life-threatening complication in newborns that might successfully be treated with methylxanthines (aminophylline, theophylline), decreasing its incidence and the need for mechanical ventilation [1,2]. Caffeine has become the drug of choice to treat neonatal apnoea due to its efficacy, tolerability, large therapeutic window, and safety margin [3]. Caffeine is a xanthine exerting many complex and pleiotropic effects on various organ systems mediated by a variety of mechanisms, including antagonism of the adenosine and GABA receptors, inhibition of phosphodiesterase enzymes, and sensitizing of various ryanodine-sensitive calcium release channels [4,5]. Its interference with the sympathetic nervous system has long been appreciated [4], although the results remain controversial [6]. As for improvement of apnoea, its effects include stimulation of the respiratory drive, enhancement of minute ventilation, increased response to hypercapnia, increased skeletal muscle tone, decreased diaphragmatic fatigue, and relaxation of airway smooth muscle [2,7]. Its effects on the cardiovascular system (CVS) are complex, including direct and indirect effects on the vessels and the heart. In the heart, it increases the heart rate (HR), cardiac contractility and stroke volume, consequently increasing cardiac output and mean arterial blood pressure [6,8,9]. As for the vascular system, caffeine exerts vasodilative effects in the pulmonary and most vessels of the systemic circulation but induces vasoconstriction in the cerebral circulation [8]. Caffeine also increases the metabolic rate, neuromuscular transmission and catecholamine release [10].

HR is modulated by an interplay between the sympathetic and the parasympathetic branches of the autonomic nervous system (ANS), and is subjected to beat-to-beat variability which can be assessed by measuring the time interval between consecutive heartbeats [11,12]. While the effect of the parasympathetic nervous system (PNS) on the HR is expressed quickly, the effect of the sympathetic nervous system (SNS) takes longer for its full expression. These different timeframes of the action of the PNS and SNS on the sinoatrial node may partly be explained by the corresponding neurotransmitter kinetics [13].

A relevant clinical tool for a non-invasive assessment of the ANS maturation in newborns, as well as the effects of the ANS on a newborn’s heart, is spectral analysis of heart rate variability (HRV) [13]. Accordingly, HRV can be separated into typical frequency-spectra, most usually a high-frequency (HF) spectrum and low-frequency (LF) spectrum, using various methods. While there is no uniform consensus on the relevant parameters of HRV that might adequately reflect the balance between PNS and SNS, one of the usually applied parameters is the ratio between the LF and HF spectra (LF/HF ratio). Although not unequivocal, higher LF/HF implies higher SNS activity, while a larger HF spectrum is suggested to reflect PNS activity [11,14,15].

In general, higher HRV is suggested to be linked to decreased cardiovascular risk and consequently decreased mortality in adults [16,17,18,19], and with well-being and decreased mortality in newborns [20,21,22]. Studies performed in newborns found an increased HRV in term compared with preterm newborns [23,24,25,26], which might point to a more mature ANS, especially the PNS branch, in terms. It has been reported that PNS develops optimally after 37 weeks of postmenstrual age (PMA; the time elapsed between the first day of the last menstrual period and birth (gestational age) plus the time elapsed after birth (chronological age) [27,28,29]. Moreover, it has been suggested that HRV in newborns is affected by sleeping position. We have previously shown an increased HRV in supine compared with prone position suggesting that supine is more favourable regarding well-being of the newborns [30].

The effect of caffeine on HRV remains controversial. Studies conducted in healthy adults mostly showed an increase in HRV after caffeine administration [31,32,33,34]. Only a few studies have assessed HRV in newborns; even less is known about the potential influence of caffeine on the ANS as assessed by spectral analysis of HRV. To this end, the aim of our study was to evaluate the impact of caffeine treatment on HRV in newborns. In addition, we investigated the potential effect of caffeine treatment on breathing frequency (BF), the HR, arterial oxygen saturation (SaO_2_), and body temperature (T). As caffeine might differently affect the ANS in newborns of various ages and as HRV is one of the few available tools for in vivo assessment of the maturity of the ANS [26], we further correlated the parameters of HRV with PMA, during as well as after caffeine treatment. We hypothesized that due to its known effects on the CVS, caffeine might affect the HRV in newborns.

## 2. Materials and Methods

### 2.1. Patients

A prospective clinical intervention study was performed in 25 haemodynamically stable newborns admitted to the Neonatal Department of the University Medical Centre Ljubljana, Division of Paediatrics due to neonatal apnoea that had been treated with caffeine citrate. The diagnostic criteria of apnoea, defined as absence of breathing for 20 s or longer or shorter accompanied by bradycardia or hypoxemia [35], applied to all our patients.

Newborns included in our study had the following (often coexisting) diagnoses: neonatal respiratory distress syndrome, mild dysmorphic features, mild non-optimal neurological signs, congenital heart defect (haemodynamically insignificant patent ductus arteriousu), newborn jaundice, congenital anomalies of the kidney and urinary tract, bronchopulmonary dysplasia, anaemia of prematurity, pneumonia, grade 1 intraventricular haemorrhage, macrosomy, cryptorchidism, hypoglycaemia, intra-amniotic bleeding, or bilateral pneumothorax. None of the listed diagnoses had any apparent impact on the haemodynamic parameters.

Exclusion criteria were severe perinatal hypoxia, infection, liver or renal insufficiency, neurological disorders, and congenital anomalies. Newborns whose data could not be used for spectral analysis due to artefacts of the recordings were also excluded from the study.

The caffeine treatment regimen consisted of a loading dose of 20 mg/kg body mass of caffeine citrate (i.e., 10 mg/kg caffeine), followed by a daily maintenance dose of 5 mg/kg of caffeine citrate (i.e., 2.5 mg/kg caffeine) after 24 h. Recordings on caffeine treatment were collected 48 h after the loading dose of caffeine. The newborns were treated for ten days on average, either orally or intravenously, according to their clinical state; after the cessation of caffeine, the newborns had no remining respiratory distress. It has previously been shown that the route of administration does not affect the pharmacokinetics of caffeine as there is almost complete bioavailability after oral or intravenous administration [36].

The study was approved by the National Medical Ethics Committee of the Republic of Slovenia (0120-458/2016-3 KME 67/09/16) and complies with the principles of the Declaration of Helsinki, the European Convention on Human Rights and Biomedicine, and the Slovenian Code of Medical Deontology. Written parental consent was obtained for all participants. Our trial was retrospectively registered on 27 April 2021, with reference number NCT04869176.

### 2.2. Study Setting

Each newborn underwent two measurement settings: the first set of experiments was performed while receiving the caffeine citrate. The second set of experiments was repeated 100 ± 26 h after the treatment with caffeine had been withdrawn. These newborns served as controls. In eight newborns, we could not perform the control measurements due to technical difficulties.

**Figure 1:**
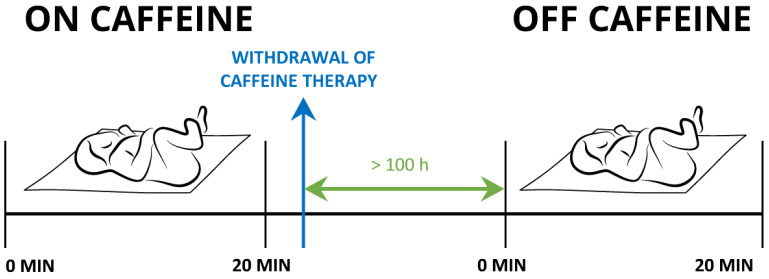
Timeline of the study protocol.

Measurements were performed while the newborns were sleeping in a supine position. Their state of alertness was scored one or two as determined according to Prechtl [37]. We simultaneously assessed each newborn’s BF, SaO_2_, T, and ECG, in a 20 min interval (Figure 1).

BF, SaO_2_ and T were measured three times during a suitable alertness state of the newborn and an average over three measurements was reported. BF was determined manually by observing the chest movement. SaO_2_ was measured by a pulse oximeter (IntelliVue MP 50, Philips, Eindhoven, the Netherlands) attached to the right hand. T was measured by a frontal non-contact infrared thermometer (Veratemp, Weston, FL, USA).

As for the ECG tracing, five precordial ECG electrodes (ECG Holter, Vision 5L, Burdick, Milwaukee, WI, USA) were attached to the newborn’s chest prior to feeding. After feeding, the newborn was placed supine in a bed and a 20 min tracing was obtained during the suitable alertness state. During the recordings, the heating was turned off to avoid potential interference with the ECG signal.

### 2.3. Data Analysis

Data were extracted from the ECG recordings using the programmes Vision Premier ver. 3.4 (Cardiac Science Corp., Waukesha, WI, USA) and Nevrokard (Nevrokard, Izola, Slovenia). For the analysis of each recording, a 5 min segment was used. Before and after an ectopic beat, RR intervals (intervals between two subsequent R-waves of the QRS complex) were measured and replaced by two interpolated RR intervals, which were calculated from a proceeding and a succeeding sinus interval. If the programme (Nevrokard) did not correctly determine the R-wave due to the artefact, the exact position of the R-wave was determined by the investigator who performed the spectral analysis. Data containing artefacts in more than 1% of the corresponding segment were removed from subsequent analyses.

Interpolated RR intervals were analysed using fast Fourier transformation, a frequency domain linear method of assessing HRV. Fast Fourier transformation enclosed 1024 points, and a Hamming window was used for the calculation of spectral density. In addition to the total power spectrum (TP), two frequency bands were assessed: one for the LF (in the range of 0.04–0.15 Hz) and one for the HF (in the range of 0.15–1.0 Hz), LF and HF being represented also as a ratio (LF/HF) and in normalized units (LFnu, HFnu) [11]. We selected a segment which corresponded to the suitable alertness state of each newborn. A mean HR value was obtained from the corresponding analysed segment.

### 2.4. Statistical Analysis

Statistical analysis was performed by Microsoft Excel 2010 and IBM SPSS Statistics 24. The data distribution was tested by the Shapiro–Wilk normality test. The numeric variables are shown either as an arithmetic mean and standard deviation (SD) for a normal (HR, BF, T), or as a median and interquartile range (IQR) for an abnormal distribution (SaO_2_, HRV parameters), respectively.

We compared variables according to the presence of caffeine (‘on caffeine’ or ‘off caffeine’).

We assessed potential correlation between PMA and HRV parameters regarding the presence of caffeine. Using univariate linear regression, we correlated the data from the first measurement (PMA ‘on caffeine’ and HRV parameters ‘on caffeine’) and, separately, for the second measurement (PMA ‘off caffeine’ and HRV parameters ‘off caffeine’).

Student’s *t*-test was used for comparisons of normally distributed variables, and the Wilcoxon signed-rank test for non-normally distributed data. The correlation between HRV parameters and PMA was tested with the Pearson correlation coefficient. The significance level was set at *p* ≤ 0.05.

## 3. Results

The demographic data and baseline characteristics of the newborns are shown in Table 1. The newborns had been treated with caffeine after being diagnosed with neonatal apnoea. The treatment was discontinued at 37 ± 2 weeks of PMA. In the subsequent analysis on the potential effects of caffeine, we included only 17 newborns in whom the measurements could be repeated after withdrawal of caffeine.

### 3.1. The Effect of Caffeine on the Heart Rate, Breathing Frequency, Arterial Oxygen Saturation and Body Temperature

The BF was significantly higher during caffeine treatment. No significant differences in the HR, SaO_2_ or T were found between the treatment (‘on caffeine’) and post-treatment (‘off caffeine’) (Table 2).

### 3.2. The Effect of Caffeine on HRV

No associations between caffeine treatment and any of the HRV parameters were found (Table 3).

### 3.3. The Correlation between Postmenstrual Age and the Parameters of HRV

We found a positive correlation between some of the HRV parameters and PMA. During caffeine treatment, we found a moderate positive correlation between PMA and LF (Pearson correlation coefficient = 0.42; *p* = 0.039), and between PMA and TP (Pearson correlation coefficient = 0.41; *p* = 0.044). After cessation of the caffeine treatment, a positive and moderately strong significant correlation was found between PMA and LF (Pearson correlation coefficient = 0.57; *p* = 0.017), and between PMA and TP (Pearson correlation coefficient = 0.5; *p* = 0.041).

## 4. Discussion

The main finding of our study is that treatment with caffeine does not affect any of the spectral indices of HRV in newborns. Moreover, caffeine did not induce any significant changes in HR, SaO_2_ or T but did increase BF. Apparently, the maintenance dose of 2.5 mg/kg body mass to treat apnoea is not sufficient to exert any measurable effects on the above parameters, except for an expected increase in BF. To the best of our knowledge, our study is the first to have assessed the effect of caffeine on HRV in newborns at 37 weeks PMA. The preterm newborns included in the only two available similar studies were significantly younger [1,9].

The only parameter that was affected by caffeine was BF. The increase in BF after the caffeine treatment was expected, as caffeine is a known stimulant of the respiratory centre.

In our study, the values of SaO_2_ were comparable during and after the cessation of caffeine treatment. As the values of SaO_2_ were mostly at their physiological limits, caffeine could have hardly induced an additional increase.

Caffeine reportedly increases the rate of metabolism and could thus potentially induce an increase in T. Nevertheless, the maintenance dose used in our study apparently was not sufficient to induce any measurable effects of caffeine on T since we found no differences in T during versus after the caffeine treatment. In fact, this is a favourable outcome regarding the interpretation of the HRV data; namely, an elevated T increases HR [38], and if this had been the case in our study, it would have interfered with the interpretation of the effects of caffeine on HRV. Moreover, increased HR implies additional burden to the heart as it significantly increases the heart work [39].

In our study, the HR and HRV parameters obtained during caffeine treatment were comparable with the parameters after discontinuation of treatment. Since cardiogenic effects described in the literature in terms of tachycardia only occur when applying toxic doses of caffeine (plasma level exceeding 20 mg/L) [40], we may conclude that our maintenance dose was not sufficient to impact the HR in newborns. Our results regarding the effect of caffeine on HR and HRV are in accordance with the study of Ulanovsky et al. who also did not observe any impact of caffeine (applied in a loading dose of 15–20 mg/kg/day, followed by a maintenance dose of 5–10 mg/kg/day) either on the HR or HRV in premature newborns [1]. Yet, their sample may not be comparable with ours, as the newborns in their study [1], as well as in that of Huvanandanas et al. [9], were younger than the newborns in our study (gestational age 30.3 ± 2.5 weeks and 27.0 (23.6–33.3) weeks, compared with 34 ± 5 weeks in our study) and also had significantly lower birth weight (1397 ± 458 g and 934 (552–2100) g compared with 2353 ± 914 g in our study) [1,9]. On the other hand, Huvanandana et al., who compared the results of the linear and non-linear analyses of HRV in preterm newborns prior to and two hours after a loading dose of caffeine, reported an increased HRV after caffeine administration when using non-linear, but not when using linear modelling, as it was analysed in our study. They suspected that linear metrics might not adequately capture potentially altered dynamics in the HR control [9]. Indeed, a lack of consistency in the analysis methods applied has already been exposed in the review by [41]. Moreover, the influences of caffeine on the heart and the activity of ANS seem controversial as caffeine has been shown to increase the HF component of HRV in adults, apparently increasing the PNS activity [31,42]. On the other hand, caffeine has been suggested to modulate the response to stress by increasing the levels of circulating catecholamines and cortisol, both of which strongly impact the heart and the sinoatrial node [4].

Although all parameters in our study were measured after the newborns were fed, and during the first 20 min of sleep, the HRV measurements could also be dependent on the sleep phase, which we did not assess. Namely, sleep onset in newborns corresponds to a REM phase. One sleep cycle lasts for about 50 min, and consists of equal subsequent proportions of REM and non-REM sleep [43,44]. Yiallourou et al., who assessed HRV in preterm and term newborns, found significantly increased values of LF/HF, and both LF and TP spectra during REM compared with non-REM sleep [24]. Similarly, Takatani et al. showed higher LF, HF and LF/HF during REM than during non-REM in newborns [25]. To this end, assessing the phase of sleep in our newborns might be valuable for further interpretation.

We found a positive correlation between HRV (LF and TP but not HF or LF/HF) and PMA, irrespective of caffeine treatment. Nevertheless, HRV is known to be dependent on mean HR—the lower the mean HR the higher the HRV [27]. In our study, the HR did not significantly change during the two measurements. A positive correlation between PMA and HRV supports the idea of ANS maturation with increasing age; however, due to the narrow time frame (100 ± 26 h) between the consecutive measurements, the results should be interpreted with caution. Our results are accordant with our previous study [30] and with the study of Sahni et al., who showed a significant increase in HRV with increasing PMA in growing low birth weight infants, and implied an important role of ANS maturation in the control of cardiac activity [45]. Based on our observations, it seems that the maturation of the ANS proceeds independently of caffeine. Contrary to our hypothesis and our observation from our previous study [30], where the study population was on average four weeks older, the HF did not significantly increase with increasing PMA in the present study. The discrepant findings could be due to limitations of the spectral analysis. In the present study, the mean HR was about 139 beats per minute. The Nyquist frequency of our sample was, therefore, 69.5 per minute, which is approximately 1.16 Hz. The upper limit of the HF spectrum used for our analysis was 1 Hz. Anything above this value was, therefore, a part of HF score that fell out of the analysis.

It is also important to note that in humans, if the BF is outside the HF band, the power of HF cannot be considered as a marker of vagal modulation. Although in the developing newborn we may expect that the PNS is still maturating, we probably should use the same rationale for the definition of the HF band. The highest BF measured in our study was 56 beats per minute (with an SD of 12), which is borderline for the upper limit of the HF band. This indicates a limitation in the use of the HF band in our sample.

In addition, the influence of respiratory sinus arrhythmia on HRV should be taken into consideration. As the PNS has a much shorter delay than the SNS on the heart to covariate with respiration, the PNS represents the main factor contributing to respiratory sinus arrhythmia. Many reports have confirmed that a considerable portion of the HRV affected by PNS actually arises from respiratory sinus arrhythmia [27,46], yet this phenomenon has not been adequately studied in newborns to date. We can speculate that the PNS, which is not fully developed in newborns, thus contributes less to HRV. It is also possible that HF did not significantly increase with PMA due to higher BF in newborns as compared with BF in adults. Moreover, our newborns presented with neonatal apnoea, which is related to a respiratory disorder or abnormal breathing patterns; therefore, their breathing pattern might have differently affected the HF than the pattern in healthy newborns; nevertheless, at least after cessation of therapy when the newborns exhibited no respiratory distress, this explanation seems less probable.

A limitation of our study is the small sample size with rather heterogenous underlying diagnoses, yet it was homogenous regarding PMA, and the haemodynamic and breathing parameters assessed. Another limitation is the rather long half-life of caffeine in preterm newborns (87 ± 25 h at 35 weeks PMA [47]). Due to its prolonged half-life, caffeine could persist up to 38 weeks PMA due to immature liver function [48]. Accordingly, caffeine concentration might still have been elevated in some newborns at the time of the control measurements. Unfortunately, we could not exceed this time frame due to the limited duration of hospitalizations; however, by ensuring that 100 h on average passed between the last dose of caffeine and the second measurement, this scenario seems rather unlikely. It would be more meaningful to assess the relationship between caffeine and HRV based on caffeine concentrations in blood rather than just on caffeine dose, yet our newborns did not have central venous access, and it would, therefore, be unethical to collect the blood samples for the purposes of our study only.

As stated above, HF values above 1 Hz could not be included in the analyses due to the upper limit of the frequency spectrum, therefore narrowing the available data.

Additional insight into potential effect of caffeine on haemodynamic parameters would be obtained by continuous measurement of arterial blood pressure, which was not performed in our study. Our study could be improved by continuous measurement of BF, as well as blood pressure monitoring, and by also measuring EEG to determine the phase of sleep, which might have impacted the outcome.

Finally, a note should be given on the study design: a more reliable estimation of the potential impact of caffeine on HRV would be obtained causally, i.e., by first assessing the parameters before caffeine was applied, and subsequently during the application of caffeine. As our newborns needed prompt treatment for apnoea, such a design was not feasible. Another approach would be to check the effect of caffeine in healthy newborns which would enable a successive regimen of measurements, yet the application of caffeine in healthy newborns is not acceptable from an ethical point of view.

## 5. Conclusions

This is the first study addressing the impact of caffeine on HRV in newborns at 37 weeks PMA. The results of our study did not show any effects of caffeine on any of the parameters of HRV, but increased the BF, as expected. We might speculate that the maintenance dose of caffeine in newborns is too low to affect the HR and HRV. Furthermore, we showed a positive correlation between HRV and PMA, regardless of caffeine intake, pointing to ANS maturation with age that apparently is not subjected to any alterations induced by a standard treatment dose of caffeine. Considering the data on the physiological effects of caffeine shown in this study, as well as in some other studies, we may conclude that caffeine at the prescribed dosage is a suitable treatment for apnoea in newborns.

## Figures and Tables

**Table 1:** Demographic data of the newborns.

	At Birth	Measurement at the Time of Loading Dose of Caffeine	Measurement While on Maintenance Dose of Caffeine	Measurement While off Caffeine
Postmenstrual age (weeks)	34 ± 5	37 ± 4	37 ± 3	37 ± 2
Body mass (g)	2353 ± 914	2659 ± 676	2786 ± 560	2745 ± 512
Head circumference (cm)	31 ± 4	33 ± 3	34 ± 2	34 ± 3
Caffeine dose (mg/kg BM/day)		9.84 (5.65–9.93)	2.55 (2.31–2.67)	
Apgar score 1 min	8.0 (7.5–9.0)			
Apgar score 5 min	9.0 (7.0–9.0)			
Body length (cm)	46 ± 7			

Data are presented as an arithmetic mean and standard deviation (postmenstrual age (PMA), body mass (BM), head circumference, body length), or as a median and interquartile range (caffeine dose, Apgar score), where appropriate. PMA is the time elapsed between the first day of the last menstrual period and birth (gestational age) plus the time elapsed after birth (chronological age) [29].

**Table 2:** Physiological variables during (‘on caffeine’) and after (‘off caffeine’) caffeine treatment.

	HR (Beats/min)		BF (Breaths/min)		SaO_2_ (%)		T (°C)	
		*p*		*p*		*p*		*p*
On caffeine	138.6 ± 12.0	1	56.2 ± 12.5	0.023 *	99 (97–100)	0.477	36.7 ± 0.4	0.332
Off caffeine	138.6 ± 13.1		50.7 ± 13.2		99 (97–100)		36.8 ± 0.3	

Data are presented as an arithmetic mean and standard deviation (HR, heart rate; BF, breathing frequency; T, body temperature), or as a median and interquartile range (SaO_2_, arterial oxygen saturation). * *p* < 0.05; Student *t*-test (HR, BF, T) or Wilcoxon signed-rank test (SaO_2_) were used.

**Table 3:** The HRV parameters during (‘on caffeine’) and after (‘off caffeine’) caffeine treatment.

	TP (ms^2^)		LF (ms^2^)		LFnu		HF (ms^2^)		HFnu		LF/HF	
		*p*		*p*		*p*		*p*		*p*		*p*
On caffeine	522 (286–1399)	0.653	219 (99–357)	0.435	63.9 (54.5–72.2)	0.868	107 (66–272)	0.523	36.1 (27.1–43.1)	0.619	1.8 (1.3–2.7)	0.877
Off caffeine	732 (228–1270)		232 (85–598)		69.1 (52.8–73)		145 (57–268)		30.9 (27–47.2)		2.2 (1.1–2.7)	

Data are presented as a median and interquartile range. TP, total power; LF, low-frequency spectrum; HF, high-frequency spectrum; LF/HF, the ratio between LF and HF spectra; N, number of newborns; *p*, *p* value; Student *t*-test (TP, LF, HF, LF/HF) or Wilcoxon signed-rank test (LFnu, HFnu) were used.

## Data Availability

The data presented in this study are available on request from the corresponding author.
